# Promoting public health legal preparedness for emergencies: review of current trends and their relevance in light of the Ebola crisis

**DOI:** 10.3402/gha.v8.28871

**Published:** 2015-10-07

**Authors:** Odeya Cohen, Paula Feder-Bubis, Yaron Bar-Dayan, Bruria Adini

**Affiliations:** 1 PREPARED Center for Emergency Response Research, Ben-Gurion University of the Negev, Beer-Sheva, Israel; 2 Department of Emergency Medicine, Recanati School for Community Health Professions, Faculty of Health Sciences, Ben-Gurion University of the Negev, Beer-Sheva, Israel; 3 Department of Health Systems Management, Faculty of Health Sciences & Guilford Glazer Faculty of Business and Management, Ben-Gurion University of the Negev, Beer-Sheva, Israel

**Keywords:** Ebola, emergency, emergency management, public health legal preparedness

## Abstract

**Background:**

Public health legal preparedness (PHLP) for emergencies is a core component of the health system response. However, the implementation of health legal preparedness differs between low- and middle-income countries (LMIC) and developed countries.

**Objective:**

This paper examines recent trends regarding public health legal preparedness for emergencies and discusses its role in the recent Ebola outbreak.

**Design:**

A rigorous literature review was conducted using eight electronic databases as well as Google Scholar. The results encompassed peer-reviewed English articles, reports, theses, and position papers dating from 2011 to 2014. Earlier articles concerning regulatory actions were also examined.

**Results:**

The importance of PHLP has grown during the past decade and focuses mainly on infection–disease scenarios. Amid LMICs, it mostly refers to application of international regulations, whereas in developed states, it focuses on independent legislation and creation of conditions optimal to promoting an effective emergency management. Among developed countries, the United States’ utilisation of health legal preparedness is the most advanced, including the creation of a model comprising four elements: law, competencies, information, and coordination. Only limited research has been conducted in this field to date. Nevertheless, in both developed and developing states, studies that focused on regulations and laws activated in health systems during emergencies, identified inconsistency and incoherence. The Ebola outbreak plaguing West Africa since 2014 has global implications, challenges and paralleling results, that were identified in this review.

**Conclusions:**

The review has shown the need to broaden international regulations, to deepen reciprocity between countries, and to consider LMICs health capacities, in order to strengthen the national health security. Adopting elements of the health legal preparedness model is recommended.

Planning for the prevention and mitigation of morbidity, mortality, and environmental damage is fundamental to public health system preparedness for emergencies (1). An essential element in this planning process is the creation of a legal infrastructure to be activated during all phases of the emergency (2) from pre-event to recovery. The existence of a legal framework is particularly important in large scale crises (3) and scenarios requiring humanitarian assistance (4).

Public health legal preparedness (PHLP) involves more than legislation. Moulton et al. (5) defined PHLP as ‘legal bench-marks or standards essential to the preparedness of that system’. Accordingly PHLP includes 1) laws/legal authorities; 2) competencies of those who apply the law; 3) information relevant for the application of the law; and 4) coordination across sectors/jurisdictions. Benjamin et al. (6) state that although these components can be broadened the improvement of legal preparedness must address all four core elements and not focus on one element (e.g. legislation) only, which will lead to an incomplete solution (5). Globalisation requires integrated, joint actions aimed to facilitate management of international threats including the use of PHLP.

The aim of this paper is to review recent theoretical and research trends regarding legal preparedness of the public health system for emergencies. In particular, the PHLP during the current Ebola crisis will be discussed.

## Methods

Two parallel literature reviews were conducted to explore 1) theoretical and research trends of PHLP in developed countries, as well as in low- and middle-income countries (LMICs), and 2) the application of PHLP during the recent Ebola crisis. The reviews were conducted from September to December 2014 and encompassed peer-reviewed articles published in English as well as reports, theses, and position papers. The study search encompassed eight electronic databases: Cochrane, LexisNexis, Proquest, PubMed, Science Direct, Scopus, Social Science Research Network, and Web of Science; the Google Scholar search engine was also employed.

### Review of theoretical and research trends of PHLP in developed states and LMICs

The keywords used to extract relevant articles were *public health*, *legal preparedness*, and *emergency*. The year of publication was limited from 2011 to 2014. However, if one of the reviewed articles emphasised findings that were based primarily on specific previous models or legislative actions, these earlier articles were also reviewed. Due to the very limited findings concerning LMICs, and in order to identify elements within the context of PHLP that are implemented in those countries, additional keywords – *regulation* and *legislation* – were introduced to the review procedure. Articles were only included in this review if they dealt with legal aspects of emergency situations. An exclusion criterion was a focus on routine issues (e.g. the obesity epidemic). This criterion was used to screen all abstracts included in the study. In the final stage, the articles were grouped by themes.

### Review of application of PHLP during the current Ebola crisis

Keywords used to extract relevant articles were *Ebola*, *public health*, *legal preparedness*, and *emergency*. The review was limited to 2014. Articles were included if they focused on legal issues regarding the Ebola outbreak. This criterion was used to screen all abstracts included in the study. In the final stage, the articles were grouped by themes.

## Results

### The role of PHLP in emergency response

PHLP plays an important role in the overall functioning of the health system during emergencies (7, 8). Providing legal assistance in the midst of a disaster is central to any response plan (9, 10). Adini et al. (11) found that following standard operating procedures in an emergency is crucial for assuring preparedness. Other aspects of legal preparedness relate to the status of volunteers and their ability to provide humanitarian aid after a disaster (12).

Orenstein (13) asserts that some laws, though appropriate for routine health activities, may hinder operations during emergencies. While declaring a state of emergency may facilitate waiving laws, the implications of such waivers must be carefully evaluated. During emergencies, as maintained by Courtney et al. (14), healthcare providers operate under challenging conditions that may require deviation from existing treatment protocols, necessitating the development of strategies to protect against legal liabilities. Similarly, Chan (15) argues in favour of granting legal immunity to private physicians to protect them against damage claims. Conversely, it is important to protect the vulnerable population from uncertified personnel performing beyond their capacity. According to Wang (4), this issue manifests itself more intensely in situations of cross-national mutual aid that are regarded as reciprocal gestures of goodwill, where programmes cross boundaries and achieve their expected goals quickly. Thus, it is important to define obligations and rights and to establish roles, items, and standards. Other researchers (5, 6) perceived the competency of health workers as one of the core elements of PHLP.

### PHLP in epidemic scenarios

Pandemic outbreaks inflict widespread suffering and may negatively impact international economic stability (16). Managing pandemics involves coordinating different aspects of the health and social systems. In such situations, the law, which is a small but crucial component of emergency preparedness (8, 17), assumes increased importance (18–20). The speed with which viruses spread makes it imperative to ensure that a legal framework is in place to delineate mechanisms for effective epidemic management, within the country itself as well as globally (17, 21, 22). Accordingly, efforts and reforms in this direction have been proposed and implemented worldwide, in developed countries as well as LMICs (17) from the United States to China (23).

According to Hodge (19), although legal reforms have occurred in the United States in the last years, three core challenges will continue to engage experts during the next decade: the legal implications of multiple emergency declarations, legal triage, and liability protection for practitioners and entities implementing crisis standards of care in response to declared emergencies (19).

Following outbreaks of severe acute respiratory syndrome (SARS) and avian flu (H5NI) in 2004–2005, the World Health Organization (WHO) adopted the International Health Regulations (IHR), whose goal is ‘to prevent, protect against, control and provide a public health response to the international spread of disease’ (24). The WHO also published a checklist designed to help countries prepare effectively for infectious emergencies (25).

The experiences of the global health system in dealing with the 2009 pandemic flu (H1N1) had a significant impact on subsequent implementation of the IHR (17, 20, 26, 27). The IHR made valuable contributions in various areas, including issuing clear-cut reports from afflicted countries, integrating information from diverse sources, and monitoring unnecessary human rights limitations (26). In addition, it has become a useful decision support tool (28). However, analysis of how the IHR are applied in LMICs reveals the challenges that still lie ahead (26). The main weakness is lack of resources and the ensuing inability to meet IHR demands and develop effective public health services (16, 17, 26, 29). In addition, the IHR do not make allowances for local and cultural variability (17, 30–32). Wilson et al. (26) note several arenas in which IHR application needs to be strengthened, including declaring a health emergency in international scenarios and developing mechanisms to improve compliance with WHO and IHR recommendations. Kool et al. (33) noted that in order to achieve the IHR goals it is crucial to simplify identification and detection capacities in countries lacking advanced health systems. For example, it is necessary to base disease definitions on clinical signs and symptoms without the need for laboratory confirmation (33).

Epidemic scenarios that involve laws and PHLP concerning isolation, quarantine, and social distancing are worthy of broad attention. Limiting individual freedom in order to protect the public health has significant implications for managing infectious diseases (19, 25, 34, 35). Preparing a legal infrastructure to administrate these situations is crucial, including declaration of an emergency situation which authorises public health officials to activate such means (19). Nonetheless, operationalising them should not be based on legal facets alone, but should rather also consider judicial aspects. Coercion may decrease the effectiveness of protection that could be achieved through voluntary compliance. It may also increase the probability of a constitutional crisis (34). Mosher (8) notes that the law's promise of protection against abuses during an epidemic offers limited space for critics worried that individual liberties will yield to national security and public health concerns. In such cases, it is important to create an alternative framework in order to voice the views of those who are socially marginalised and have been largely silenced (8).

### The balance between individual rights and the common good

A central aspect of PHLP is balancing individual needs and the common good. The need to protect individual freedom arises in all types of emergency situations, but has significant implications for the management of infectious disease outbreaks (25, 34). Turnock (36) describes two major aims of public health laws: to protect and foster public health and to safeguard the rights of the individual. In emergency situations, imposing limitations on individual rights is unavoidable (34). In the United States, in the 1905 Supreme Court case *Jacobson v. Massachusetts* the court sustained the right of the authorities to use penalties to pressure people to be vaccinated during a smallpox epidemic. This ruling interpreted the use of the public health authority and the way the court balanced two strong competing values: the public good and individual liberties (37). Gostin (35) argues that the resulting ethical conflict is more acute in the period preceding the emergency. However, early legislation enables legal definition of individual rights, thereby facilitating optimal actions during the emergency itself (35). According to Gerwin (34), during pandemics, governance that assures a legal balance between the needs of the public and those of the individual enhances public trust in the authorities’ ability to manage the situation. By contrast, in the wake of the 2001 terror attack in the United States, the rights of the individual were significantly curtailed by a powerful government supported by legal measures (8).

### PHLP in LMICs

Limited information was found regarding legal preparedness for emergencies in LMICs. Most sources focused exclusively on infectious disease management while only few related to legal aspects in the wider context of health system operation. Fischer and Kats (32) mention the rural-to-urban migration phenomenon that produces ‘mega-cities’ of 10 million or more inhabitants. According to United Nations estimates, three-quarters of the mega-cities are located in LMICs. This global trend exposes populations to disaster vulnerabilities and is associated with a dearth of risk management infrastructures – physical, governmental, and legal (32). Nishtar et al. (38), analysing the Pakistani health system, stated that one of its strengths is its legislative activism and the federal structure of the government, which promote the health system's ability to overcome challenges. According to them (38), it is important to establish laws and regulations governing public–private interactions, insurance, and e-health and thus contribute towards a coordinated, joint preparedness. Van Niekerk et al. (39) found in studying South Africa that certain laws enacted for disaster risk reduction are not implemented due to lack of funds and that laws need to be updated to ensure coordination between public and private sectors.

Globally, LMICs play a crucial role in promoting prevention measures and immunisation programmes against diseases (40). Kaddar et al. (40) contend that the focus should be on middle- rather than low-income countries. Regarding immunisation, vaccines are designated by the WHO according to the population's needs, supported by the Global Alliance for Vaccines and Immunization (41).

An additional aspect of the legislative infrastructure relates to counterfeit medicines. Despite the efforts of the WHO (42), the regulatory structures prevailing in LMICs cannot cope with the problem of counterfeit drugs and their use (43).

### PHLP in developed countries

Health legal preparedness is more prevalent in developed countries compared to LMICs. Most publications dealing with PHLP focus on the creation of legal conditions (e.g. emergency declaration, legal immunity) which may promote public health preparedness for emergencies, rather than implementation of international regulations.

While the increasing importance of PHLP is a worldwide trend, it is especially well developed in the United States. The first call to address the issue of health system legislation was published by the Institute of Medicine in 1988 (44). This call was heeded after different emergency events that occurred during the first decade of the twenty-first century (the World Trade Center attack in 2001, the mailing of envelopes containing anthrax, the SARS epidemic in 2003, and Hurricane Katrina in 2005) (45). These natural disasters and man-made events significantly impacted US society and government (35). In addition, they demonstrated the need for a policy that would include, *inter alia*, a legal response defining how the health system should manage such scenarios. A health legal preparedness model was developed and adapted by the US legal and health systems. It encompasses four core components (5, 46): 1) law – the authoritative infrastructure of public health bodies that activate the public health system, 2) competencies – qualification of experts in areas common to legislation and health preparedness, 3) information – updating and publicising health laws for content experts and healthcare workers and 4) coordination between legal systems and within emergency response agencies. Current publications dealing with this model discuss researching and expanding its components (45–47), as well as broadening its application to situations that the health system faces routinely (44).

Current US literature reflects the recent improvements in the PHLP field. Studies dealing with preparedness are designed to reveal and resolve ethical and legal dilemmas in order to promote an optimal response for future situations. In addition to extensive coverage of legal aspects dealing with pandemic scenarios (3, 19, 34, 48, 49), publications also consider the impact of PHLP on issues such as legal triage (10), motivating health workers during emergencies (2, 48, 49), human rights during crises, legal coordination of relevant emergency bodies (10), and the effect of an emergency declaration on health system management (2, 50). Hodge (19) notes that upon declaration of a public health emergency the Model State Emergency Health Powers Act (2001) authorises public health officials to undertake a set of activities dealing with early detection, as well as to care for and protect public health, treat exposed or infected persons, and seek out-of-state volunteers. The declaration is defined to inform the population, through the media, using language that is cross-culturally accessible and understandable (19). All the items mentioned above reflect the creation of a legal infrastructure targeted for enhancing the US public health system.

In the European Union, emphasis is placed on routine application of a ‘no border’ policy, with minor references to emergencies (51–53). European Union law is based on legislation (Directives and Regulations) and decisions of the Court of Justice, which intervenes when the meaning or implementation of the legislation is unclear or fails during implementation (54). In the context of emergency preparedness, references to legal aspects focus on management of communicable disease outbreaks (21, 54, 55). Greer et al. (54) claim that the European Union's laws play a dominant role in safeguarding the population's health, as their common laws and regulations significantly affect the health system. Nonetheless, due to factors stemming from the complexity of the European Union and the variability that exists among the European states’ health systems, decisions are interpreted very generally with apparently vague provisions in the treaties. As a result, legislative interventions sometimes fail to adequately promote public health. For example, Hatzianastasiou et al. (56) found that Greek laws are aligned with accepted practices of international law in the context of communicable diseases in terms of safeguarding individual rights, but exhibit a lack of coherence, clarity, or systematisation and are often perceived as incomprehensible (56).

### Research in the field of PHLP

In order to understand PHLP, it is vital to review studies which cover diverse types of legal management. Although this topic could be significantly broadened, the description of current trends in PHLP would be incomplete without referring to this issue. Studying PHLP during emergencies is complex, as there is a need to refer to diverse types of knowledge such as that concerning governance, policy, and perceptions of emergency professionals in order to provide meaningful insights (4).

Legal preparedness shapes the health system and its emergency response in different ways. As such, there are various methodologies that prove efficient in studying these mechanisms. The integration of legislation into emergency management still seems to be a relatively neglected area (45). According to Jacobson et al. (45), this is due to the lack of recognition that information about public health laws promotes best practices during emergencies. Fox (55) noted two methodological concerns regarding research on health policy governance and diseases. The first concerns the researchers’ definition of *governance*, which influences what information they obtain and how they assess it. The second is that governance determines how diseases are conceptualised in order to make and implement policy.

Burris et al. (57) classify three types of health laws: infrastructural laws, intervention/implementation laws, and secondary legislation. Most studies in the field of law and health relate to intervention and secondary legislation and only a few relate to infrastructure and its effects on public health (52). Differences were found in the type of studies conducted in LMICs versus developed countries. In the former, the majority of studies focused on the effectiveness of infrastructural laws, international regulations, and the need for more flexible regulations (30, 58). In developed states, studies focused on internal legislation, legal issues that might arise during emergencies, and advanced planning for future challenges (2, 19, 56).

Most studies relating to developed countries were conducted in the United States. Differences among the individual states regarding the statutory/judicial system and the structure of healthcare agencies make it possible to investigate the effects of public health laws on the population's health. On the other hand, the variance that exists among the states influences knowledge management, which in turn affects health knowledge implementation within the framework of public health laws (59). Rutkow et al. (2) analysed differences between individual states in an effort to identify laws that impact the public health workforce and willingness to respond to emergencies. These differences can be well noted concerning emergency management, such as ensuring accessibility of the public to information via media that is influenced by cultural characteristics as defined in the Model State Emergency Health Powers Act (2001) (19).

Reviewing the studies relating to PHLP which were conducted in the last few years indicated that declaration of an emergency situation by the authorities was an essential component of emergency response and provided the health sector with flexibility and guidance concerning response parameters (50). Other researchers studied perceptions regarding public health laws among organisations involved in managing emergencies. Jacobson et al. (45) found a gap between experts’ perceptions of these laws and their basic aims, leading to severe deficiencies in health system preparedness. Public health and disaster management professionals may differ in their understanding of the law (47), which hampers their ability to cooperate effectively during emergencies. According to Kaufman et al. (7), staff training is the key to bridging differences in perception between public health workers and legal advisors. Other studies dealt with legal means for motivating healthcare workers and offering enhanced legal protection against liability while reducing the incidence of harm claims during disasters and pandemics (48, 49). To deal with the lack of familiarity with legal preparedness, multi-professional panels were created to reach consensus concerning relevant issues (60, 61). In both developed and underdeveloped countries, researchers that investigated regulations and laws that activate the health system during emergencies found inconsistencies and lack of coherence (56, 62, 63).

### The Ebola crisis: a case study of legal preparedness during a worldwide outbreak

The Ebola outbreak clearly illustrates the involvement of legal preparedness and response during an international crisis. This section will briefly review legal issues that demonstrate the different facets of the PHLP.

The re-emergence of Ebola in 2014 in West Africa, followed by the evacuation of stricken Western healthcare workers to the United States, captured the world's attention (64, 65). The fatality rate of the Ebola outbreak ranged from 36 to 74% (66). Affected countries are characterised by limited resources and political instability, with low capacity health systems and a lack of essential equipment and personnel. The probability that the Ebola virus will take root in a high-resource country is small (64, 65, 67).

The Ebola crisis displayed the importance of legal preparedness for emergency situations from a global perspective, presenting ethical and legal dilemmas of affected and unaffected countries. Managing such a crisis necessitated a multi-dimensional response, including effective functioning of health systems; coordination of diverse disciplines; international distribution; use of experimental drugs and medical procedures; safeguarding human rights; and consideration of implications in the health, political, and economic arenas. In addition, the Ebola crisis brought to the forefront basic dilemmas concerning responsibility and reciprocity among developed states and LMICs. It was essential to integrate the legal dimension into the global response in order to maximise strategies aimed at coordinating joint efforts to contain the Ebola epidemic and save lives (68).

The global response to the Ebola outbreak was insufficient (69, 70). Gostin and Friedman (70) argue that the outbreak uncovered a failure in global health leadership and that the WHO should be the global health leader. However, its budget is not commensurate with its responsibilities. As a result, some countries departed from the WHO directives and responded with excessive severity (e.g. imposing mandatory travel bans). In addition, several contaminated states could not realistically implement WHO recommendations and thus did not show full compliance with the guidelines (70).

Hodge et al. (67) note four issues that should be considered when creating legal preparedness for the Ebola crisis: health workers’ willingness to respond, experimental drugs and medical procedures, social-distancing measures in medical settings, and potential liabilities of healthcare workers and entities. Addressing these issues could help mitigate fears, improve the public health response, protect the safety of healthcare workers and communities, and promote comprehensive medical and public health services (67).

Effective management of emergencies such as the Ebola outbreak depends on the health systems’ capacities (71). Therefore, the most effective way to curb such outbreaks is to strengthen weak health systems and infrastructures (69, 71, 72). Considering the vast differences between developed countries and LMICs, immediate and extensive assistance should be provided (72). Regarding the high mortality rate of healthcare workers in the Ebola crisis (73), Hodge et al. (67) noted that the infrastructures must offset the risks taken by frontline personnel with a commitment to their protection. This issue came to the forefront because Western healthcare workers were evacuated, while frontline workers from affected places were not. In addition, the commitment to protect healthcare workers has to be standardised legally in affected countries as well as in ascending countries.

In addition to the above, a central legal issue highlighted by this crisis is protection of human rights. The acute nature of the recent outbreak necessitated imposing quarantine, isolation, and other restrictive measures, in addition to monitoring movement of travellers. However, these measures were applied excessively and, rather than proving beneficial, caused under-reporting of diagnosed patients, lowered trust in the government, and sharpened economic, political, and social challenges (68, 70, 74, 75). During this crisis, human rights violations were wide-ranging, including blockage of rural areas in Sierra Leone by the army, shooting of people who unlawfully entered Liberia from Sierra Leone, and broad-sweeping barricades in Liberia that prevented access to food, medicine, and life-sustaining services. Limiting travel from affected countries is contrary to WHO guidelines (68). Rothstein (76) noted four ethical principles that should be considered in the process of deciding whether quarantine is needed: necessity, effectiveness, and scientific rationale; proportionality and minimal infringement; humane supportive services; and public justification.

Regarding experimental treatments, it is important to formulate and adhere to ethical rules (64, 67, 69) in order to mitigate inadvertent damage, which could worsen already strained relationships between health professionals and their patients (64).

The factors mentioned above significantly affect the public's trust in responding agencies and governance systems activated during crises such as the Ebola outbreak. The relationships between international health organisations are thus impacted (70) in both underdeveloped (32) and developed states (75, 77).

## Discussion

This study reviews current trends regarding PHLP for emergencies. Over the past decade, in order to improve disaster planning and response (78), PHLP has steadily grown in importance (18). However, while legal preparedness is important in diverse types of emergencies, most of the literature focuses exclusively on pandemic scenarios. Furthermore, the status of PHLP is influenced by the characteristics of the country involved.

IHR represent a potentially revolutionary change in global health governance (26, 79). One criticism is that the regulations fail to make due allowance for local conditions and characteristics (7, 30–32). In order for IHR to be appropriately realised, health organisations must create conditions that enhance the capacities of countries in need. Regulatory attention paid to health systems’ capacities may have a positive economic consequence: the provision of structured support during the pre-crisis period would significantly reduce post-outbreak outlays for assistance (39). According to McCloskey et al. (80), there is a need to develop trust and nurture effective, meaningful collaborations between countries to facilitate rapid detection of potential pandemics and initiation of public health actions.

From this perspective, international regulations need to standardise the implementation of legal activities, motivated by a concern for global public health and well-being. Coordination of such activities would promote routine inter-state assistance as well as collaboration during emergencies. Thus, allowing the development of responses adapted to different countries’ capacities without losing sight of the overall public health goals would help expand the capabilities of poorer countries.

### The Ebola crisis

The Ebola crisis reveals the importance of creating legal preparedness that takes into account the needs and capacities of both affected and unaffected countries (67, 69, 71, 72). Inadequate capacities create severe stress on a country's ability to deal with the crisis. As a result, first and foremost, medical care is harmed. Moreover, it inflicts a severe blow to individual rights. Another facet of ethical and legal implications which was evident in the current crisis concerns the protection of health workers. This topic impacts both affected countries and the countries that provide assistance.

Reciprocity between developed countries and LMICs is a global concern targeted to protect the world's health. The current global situation and the health status in developing countries directly affect the health of the world's population. Thus, developed countries have an interest, beyond their responsibility, in catering to the health status of countries that lack vital means and resources. International legal preparedness, which considers the needs and capacities of different countries, will improve the assistance provided to countries that need it and regulate cooperation between the various stakeholders.

### Implementation of the PHLP model

The US PHLP model was intended to strengthen health system preparedness for routine scenarios as well as emergencies (5, 18). Implementing the four core elements (law, competencies, information, and coordination) (5) may increase legal preparedness by addressing the response of local legal and health systems for emergencies.

The adoption of elements of the US model by developing countries, may promote the capacities of health systems’ preparedness, which, in turn, will contribute to the increase of global health security. Developed countries have an interest in assisting the local health capacities in LMICs. International regulations incorporating these elements would in effect expand the resources available for coping with local and international public health emergencies. At present, resources are provided to underdeveloped states mainly to manage public health threats that affect developed countries – even though other public health hazards may pose a greater threat to the LMIC (26).

The challenges identified in LMICs in the course of the Ebola crisis should be studied in order to assist the tailoring of appropriate legal preparedness in these countries. In certain countries, such as the United States, laws regarding infectious diseases provide the legal framework for health system operations in routine situations as well as during emergencies (81). It is vital to understand the ways in which less developed countries manage epidemics and translate this into a legislative framework that can promote the effectiveness of local health systems.

### Research in the field of PHLP

Efforts to improve health preparedness must be supported by adequate research. Although a relatively new field, public health law provides important contributions to policy making and, by extension, to the health of the population (52). The dearth of studies in this area indicates a gap that needs to be filled. Systematic research employing advanced techniques and sensitive data analyses can facilitate the study of legal preparedness and help elucidate the causal relationship between legal reforms and emergency preparedness of healthcare systems. The findings will help promote emergency preparedness in every country.

### Limitations

This article provides highlights of current trends regarding PHLP as reflected in the professional literature. Nonetheless, this study did not fully examine legislation in the investigated countries. The keywords used in the literature search process did not include the word *disaster*. Nevertheless, in checking the articles that were found using the search engines, researchers noted only one article that was not included in the findings based on the term *emergency*. In addition, the literature review only included papers in the English language, which might have excluded publications from non-English speaking countries. This article provides an overview of various issues regarding PHLP but does not encompass all the particulars.

## Conclusions

The role of the legal component in building emergency health preparedness is gaining increasing recognition worldwide, although in many countries this has been expressed only in the context of infectious diseases. The Ebola epidemic revealed that despite adoption of international regulations by LMICs their health systems still lack the capacity to manage such epidemics. There is a need to boost effective implementation of international regulations by these states, thereby strengthening their ability to deal with routine and emergency situations and fostering global health security. The IHR present a good starting point, although additional work is needed to find a legal framework that will strengthen the willingness of the various stakeholders with different interests to cooperate and coordinate health preparedness programs. It is recommended that the components of the PHLP model be widely adopted as a comprehensive basis for promoting legal preparedness in local health systems, backed by sophisticated methods of analysis directed at elucidating the effect of PHLP on the capacity to cope with emergencies and disasters. Further studies are needed in the context of natural or man-made humanitarian disasters.
